# Herpes simplex infection of the eye: an introduction

**Published:** 2020-03-30

**Authors:** Bhupesh Bagga, Anahita Kate, Joveeta Joseph, Vivek Pravin Dave

**Affiliations:** 1 Consultant Ophthalmologist: Cornea Institute, L V Prasad Eye Institute, Hyderabad, India.; 2 Consultant Ophthalmologist: Cornea Institute, L V Prasad Eye Institute, Hyderabad, India.; 3 Microbiologist: Jhaveri Microbiology Centre, L V Prasad Eye Institute, Hyderabad, India.; 4 Consultant Ophthalmologist: Smt. Kanuri Santhamma Centre for Vitreo Retinal Diseases, L V Prasad Eye Institute, Hyderabad, India.

**Herpes simplex virus has affected two-thirds of the global population at one time or another. It can affect the eyes when the patient is first infected, or years later when the latent virus is reactivated.**

According to the World Health Organization (WHO), more than 3.7 billion people under 50 years – 67% of the global population – have been infected with herpes simplex virus at some point in their life.^1^ Herpes simplex infection can be particularly severe in patients who are immunodeficient, e.g., those with acquired immune deficiency syndrome (AIDS).

Herpes simplex virus is categorised into two distinct types: HSV-1 and HSV-2.

- HSV-1 is transmitted via direct contact, usually via saliva, and commonly presents as cold sores or fever blisters. It is also a cause of eye infection and significant visual impairment.^2^
- HSV-2 is transmitted via sexual contact or from mother to child during birth (neonatal herpes simplex infection). It causes genital herpes infections and, occasionally, ocular neonatal infection.

Herpes simplex virus is usually acquired in childhood or adolescence. After the initial infection, the virus can enter nerve cells in the dorsal ganglia and lie dormant, or latent.

**Primary ocular herpes infection** is often asymptomatic. The virus can present with cold sores or fever blisters (vesicular dermatitis – see Figure 1), follicular blepharo-conjunctivitis, superficial punctate keratitis (SPKs) and/or dendritic ulcer. Treatment is controversial as it is often self-limiting, but topical and systemic antivirals have been used. Aciclovir eye ointment 3% five times/day or ganciclovir 0.15%, 5-times daily for 7 days, and then three times daily for a further 7 days is recommended for dendritic ulcers.

**Recurrent ocular herpes infection** is due to activation of latent herpes virus in the nerve cells (for example, the trigeminal ganglion), usually in response to a ‘trigger’ such as fever or stress. Clinically, ocular infection can be subdivided into keratitis, uveitis and retinitis, each of which will be discussed in this article.

## Herpes simplex keratitis

This can manifest as^3^:

- Infectious epithelial keratitis
- Neurotrophic keratopathy
- Stromal keratitis
- Endotheliitis

### 1. Infectious epithelial keratitis

**Clinical features.** The earliest presentation is corneal vesicles which later coalesce to form a dendritic ulcer. The dendritic ulcer (Figure 2) presents as a branching linear lesion with terminal bulbs. The base of the ulcer stains with fluorescein while the margins (virus-infected epithelial cells) stain positively with Rose Bengal stain. When these ulcers enlarge, they appear geographical (Figure 3) with well-defined, scalloped margins.

**Diagnosis.** This is based on typical clinical signs. Occasionally, it may be necessary to confirm the diagnosis with a sample taken from a corneal scraping which is sent for virological examination, cell culture or polymerase chain reaction (PCR).^4^

**Treatment.** The usual treatment recommended is topical aciclovir eye ointment 3% five times/day until the ulcer has healed. Trifluridine is an alternative that is used in the USA. Topical ganciclovir 0.15% five times/day is an alternative treatment. If there are many recurrences, consider prescribing oral aciclovir 200 to 400 mg twice/day as prophylaxis. This drug is considered safe for long-term use with 6-monthly evaluation of renal function tests.

*Figures 1 to 9 show different clinical presentation of ocular HSV infection.*

**Figure 1:**
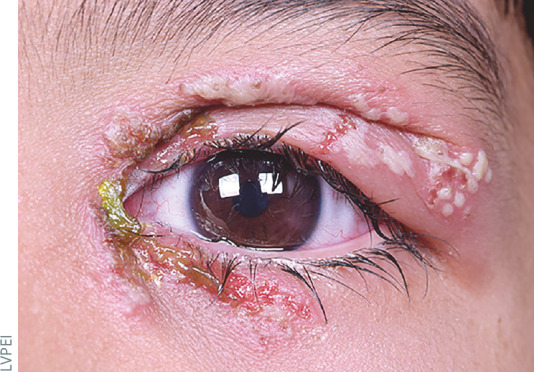
Blepharo-conjunctivitis

**Figure 2:**
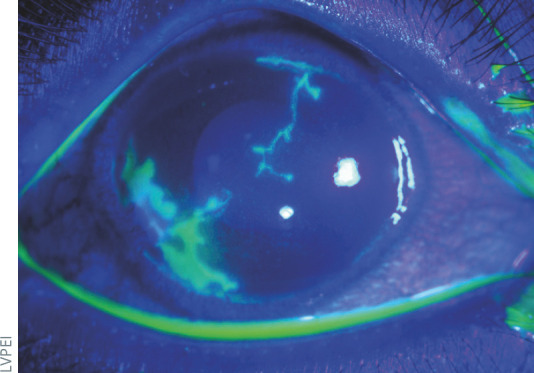
Dendritic ulcer

**Figure 3:**
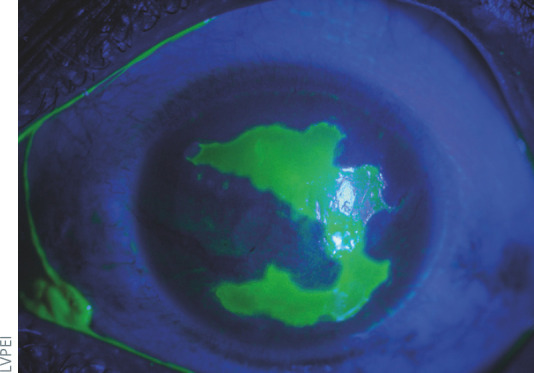
Geographical ulcer

**Figure 4:**
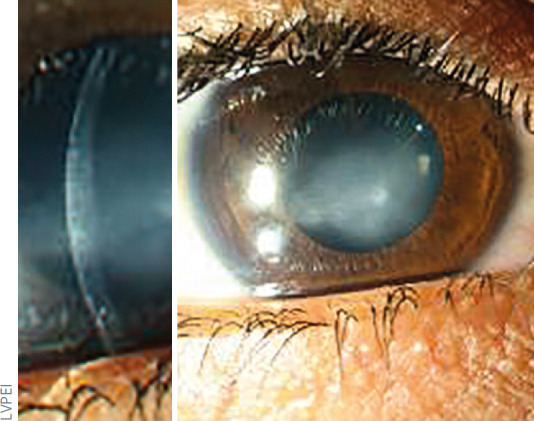
Immune stromal keratitis

**Figure 5:**
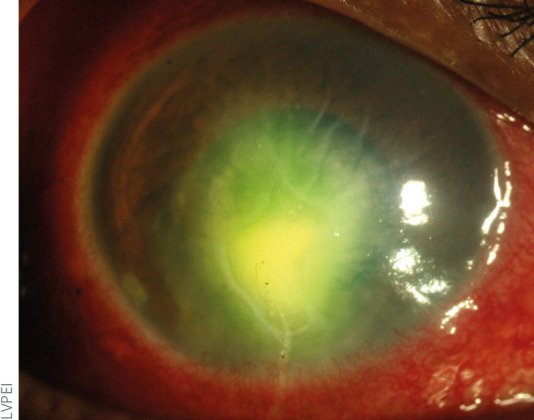
Necrotising stromal keratitis

**Figure 6:**
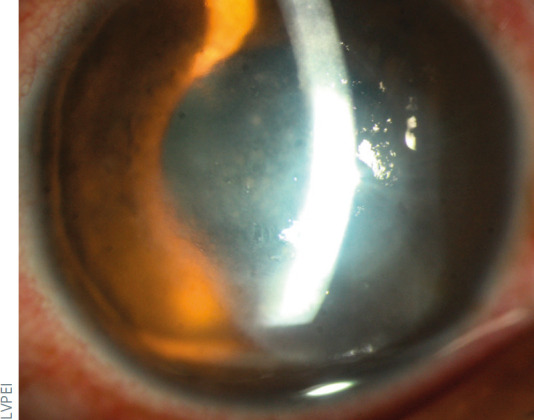
Disciform endothelitis

### 2. Neurotrophic keratopathy

**Clinical features.** There is impaired corneal sensation with a superficial punctate keratitis that can form an epithelial defect with a smooth margin (in contrast to the scalloped margins of a geographical ulcer). A grey-white stromal infiltrate (neurotrophic ulcer) with well-defined, heaped-up epithelial margins may develop; this can lead to progressive stromal thinning, corneal perforation or secondary infection.

**Diagnosis.** Microbiological evaluation with bacterial culture is recommended to rule out secondary infection.

**Treatment.** Intense lubrication and prophylactic antibiotics are recommended. The antibiotics should be kept to a minimum to reduce toxicity to the epithelium. Antivirals are not indicated. Options for non-resolving cases include bandage contact lens or amniotic membrane grafting with tarsorrhaphy if the expertise and resources are available.

### 3. Stromal keratitis

There are two main types of inflammation of the corneal stroma due to herpes simplex virus.

- **Immune stromal keratitis** presents with corneal stromal oedema (Figure 4) and folds in Descemet's membrane. This is associated with fine keratic precipitates (KPs), limbitis and (often) raised intraocular pressure (IOP); there is no epithelial defect.
- **Necrotising stromal keratitis** is due to active viral infection within the cornea. It presents with an epithelial defect (Figure 5) and dense stromal infiltration. If the infection is close to the limbus, then the marginal keratitis shows stromal infiltration and associated vascularisation.

**Diagnosis.** The diagnosis of immune stromal keratitis is made clinically, and no investigations are required. The diagnosis of necrotising stromal keratitis can also be made clinically. For confirmation, the virus can be detected in a corneal scrape using cell culture, indirect immunofluorescence (IFA) or PCR. It is important to also exclude secondary bacterial infection.

**Treatment.** This depends on the type of inflammation. **Immune stromal keratitis** is managed with topical low-dose corticosteroids 4–6 times/day with gradual tapering for 4–6 weeks, along with either topical aciclovir ointment 5 times/day or topical trifluridine for 2–3 weeks. For recurrent cases, provide prophylactic cover by giving oral aciclovir 200–400 mg 2 times/day.

**Necrotising stromal keratitis** is treated using 400–800 mg oral aciclovir 5 times/day. After complete healing of the epithelial defect, topical corticosteroids may be added to reduce inflammation, but only with regular slit lamp examination to look for any recurrence of infection or corneal thinning, which could lead to corneal perforation.

### 4. Endotheliitis

Endotheliitis is presumed to be immunogenically mediated, but the presence of live HSV has been postulated in some cases.

**Clinical features.** Endotheliitis presents with stromal oedema, similar to immune stromal keratitis (Figure 6) with keratic precipitates (KPs) limited to the area of corneal edema. The endotheliitis can be linear (starting from the corneo-scleral junction and spreading centrally), disciform (usually in the centre of the cornea), or diffuse.

**Diagnosis.** The diagnosis is made clinically.

**Treatment.** Low dose topical corticosteroids are recommended with prophylactic topical or oral aciclovir for 2 weeks.

**Figure 7:**
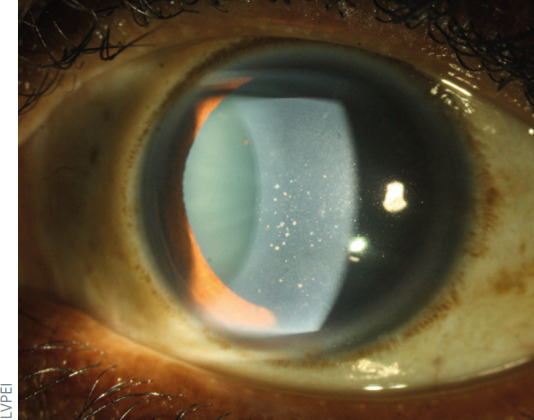
Large pigmented keratic precipitates

**Figure 8:**
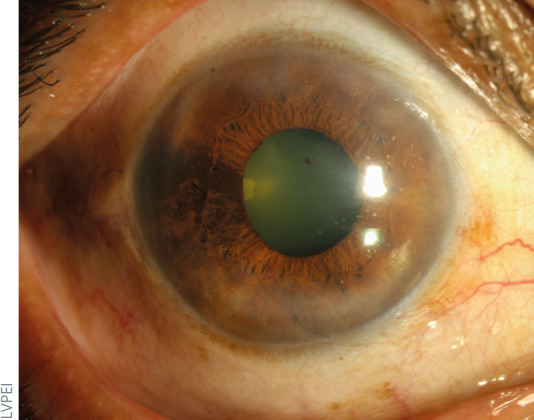
Patchy iris atrophy seen in chronic herpetic kerato-uveitis

**Figure 9:**
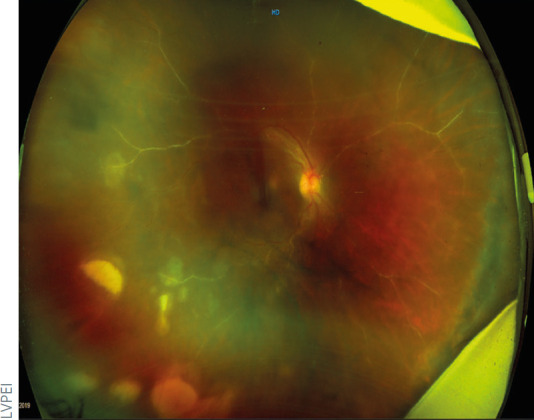
Acute retinal necrosis due to herpes simplex virus: multiple peripheral retinitis patches, sclerosed arteries, arteriolar sheathing in periphery

## Herpes simplex uveitis

Herpes simplex-associated anterior uveitis accounts for 5–10% of all uveitis cases.^5^ It is more common in older age groups, almost always unilateral and is associated with raised intraocular pressure (IOP). Pathogenesis may be due to direct infection by the virus or an immune response.

**Clinical features.** There are five clinical features which, seen together (not in isolation), characterise HSV anterior uveitis^6^:

- Recurrent episodes of unilateral anterior uveitis
- Past history of herpes simplex infection
- Raised IOP
- Diffuse KPs (Figure 7)
- Patchy or sectoral iris atrophy (Figure 8)

**Diagnosis.** Diagnosis can be confirmed by an anterior chamber (AC) tap and PCR for herpes simplex virus, or the Goldmann–Witmer coefficient (GWC) test . The GWC is a test that compares the levels of intraocular antibody production to that of serum, as measured by enzyme-linked immunosorbent assay (ELISA) or radioimmunoassay.

**Treatment.** Management includes oral aciclovir 400–800 mg, 5 times/day combined with topical steroids and topical cycloplegics. Raised IOP is treated using anti-ocular hypertensive therapy. If there is any active keratitis, topical steroids should be used with caution.

## Herpes simplex retinitis

Herpes simplex retinitis is caused when the virus infects the retina. It is seen more commonly in immunocompromised patients. It can occur in neonatal herpes simplex infection in association with herpes encephalitis. Some cases of acute retinal necrosis (ARN) are caused by the virus.

**Clinical features.** There are large, white retinal infiltrates, sheathing of the retinal vessels (Figure 9) and inflammatory cells in the vitreous (vitritis). On healing, there are large areas of scarred (atrophic) retina.

**Diagnosis.** Vitreous or aqueous tap for PCR to confirm presence of viral DNA.

**Treatment.** On diagnosis, promptly start the following treatment regimen in order to limit disease progression: antiviral treatment with intravenous aciclovir (5–10 mg/kg every 8 hours) for 5–10 days; followed by oral aciclovir (800 mg, 5 times/day) for 4–6 days. Retinal detachment following acute retinal necrosis (ARN) can be managed with a pars plana vitrectomy and silicone oil tamponade.^7^,^8^
